# Semen Quality of the First and Second Ejaculates Collected from Breeding Inactive Stallions after Cooling and Freezing

**DOI:** 10.3390/vetsci10030173

**Published:** 2023-02-21

**Authors:** Giorgia Podico, Kianna M. Spencer, Humberto B. Magalhaes, Igor F. Canisso

**Affiliations:** Department of Veterinary Clinical Medicine, College of Veterinary Medicine, University of Illinois Urbana-Champaign, Urbana, IL 61801, USA

**Keywords:** cryopreservation, sexual rest, breeding soundness evaluation

## Abstract

**Simple Summary:**

To determine the semen quality after cooling and freezing, the first and second ejaculates of the season were collected 1 h apart. After collection, the gel-free semen volume, concentration, total number of sperm, and sperm morphology were determined. An aliquot of the ejaculate was extended and cooled for 48 h; a second aliquot was processed, centrifuged, and then cooled for 48 h; and a third aliquot was processed and then frozen. The sperm parameters were assessed pre-(0 h), 24 h, and 48 h post-cooling and before and after freezing. The first and second ejaculates of the season collected 1 h apart varied in quantity but not in quality. Centrifugation prevented a reduction in the sperm quality after cooling. The semen freezing did not vary between the first and second ejaculates.

**Abstract:**

This study aimed to assess the semen quality after the cooling and freezing of the first and second ejaculates of the season, which were collected 1 h apart. After collection (n = 40 ejaculates), the gel-free semen volume, concentration, total number of sperm, and sperm morphology were determined. An aliquot of each ejaculate was extended and cooled for 48 h; a second aliquot was cushion-centrifuged and cooled for 48 h; and a third aliquot was processed and then frozen. The total motility (TM) and progressive motility (PM), plasma membrane integrity (PMI), and high mitochondrial membrane potential (HMMP) were assessed pre-(0 h), 24 h, and 48 h post-cooling and before and after freezing. The second ejaculate had a lower gel-free semen volume (*p* = 0.026). The sperm concentration was greater in the first than in the second ejaculate (*p* < 0.001). The sperm morphology was similar between the ejaculates (*p* > 0.05). Cushion-centrifugation prevented a reduction in the TM, PM, and PMI over time (*p* < 0.05). The TM, PM, and PMI decreased after freezing but not between the ejaculates (*p* > 0.05). The first and second ejaculates of the season, which were collected 1 h apart, varied in quantity but not in quality after cooling and freezing.

## 1. Introduction

Breeding inactivity is considered detrimental to equine semen quality, particularly early in the physiological reproductive season (from late winter to early spring, i.e., January to March in the Northern Hemisphere) [1,2]. Yet, it is common practice in the equine breeding industry to collect, ship out, or freeze the first ejaculates of the season from breeding inactive stallions. The reasons for processing and using ejaculates from breeding inactive stallions include a lack of knowledge or evidence concerning the effects of breeding inactivity on semen quality after cooling and freezing. Although the interaction between breeding inactivity and semen quality is seemingly stallion-dependent rather than unquestionably attributable to all stallions [3,4], the semen quality of breeding inactive stallions has been studied during the depletion of the extragonadal reserves [4]. Furthermore, the semen quality after the cooling and freezing of the first ejaculates after a prolonged period of breeding inactivity remains not well studied. 

Previously, semen cooling and freezing were not recommended for breeding inactive stallions because “old rusty” sperm was thought to not cryopreserve well; rather, the recommendation was to perform serial cleanout collections or daily collections for a week [5,6]. Despite this, breeding inactive stallions are often presented on short notice to breeding farms or veterinary practices for semen collection, cooling-shipping to breed mares with peri-ovulatory follicles, or semen freezing. This scenario may be further complicated during a showing, eventing, or racing season, which could be on or off the physiological equine reproductive season. Thus, often there is no time to collect and discard the first ejaculates of the season, either because the horse caregiver (owner, farm manager, or horse trainer) refuses to pay for cleanout semen collections (i.e., collections used for semen evaluation and to remove “old rusty” sperm stored in the extragonadal reserves of the stallion’s reproductive tract) and the associated fees or because there is no effective time to perform cleanout semen collections between sport/show events or training. Moreover, in some breeding operations, the stallions’ extragonadal sperm reserves are not emptied at the beginning of the breeding season according to current recommendations. In addition, for popular stallions, some farm managers not only do not perform cleanout collections but also freeze leftover semen from the first ejaculates of the season if the stallions must be collected for semen cooling and shipping.

Semen centrifugation is routinely used in clinical practice for the removal of seminal plasma and the concentration of sperm prior to cooling-shipping, freezing, or deep-horn insemination after cooling [6,7,8,9]. Removing the seminal plasma increases the sperm motility parameters and the longevity of the cooled semen collected from active breeding stallions [7,8,9]. Previously, traditional/conventional centrifugation was widely used over cushion-centrifugation; however, in recent years, cushion-centrifugation has become a new standard in equine practice, as it allows for sperm recovery greater than 95% with no detrimental effects on the sperm quality [7,8,9]. In the United States, semen centrifugation is not routinely applied before semen cooling and shipping unless the stallion has a low sperm concentration (<100 million sperm/mL), a history of poor semen cooling ability (i.e., a drastic decline in sperm motility after cooling), or urine contamination [10,11,12,13]. Conversely, in other countries, centrifugation is routinely used in almost all stallions before semen cooling and shipping [14]. Centrifugation is also used to process semen from breeding inactive stallions before cooling and shipping; however, there is no evidence that semen centrifugation improves the longevity and quality of the semen harvested from breeding inactive stallions. This study aimed to assess the semen quality of breeding inactive stallions’ semen collected 1 h apart after cooling and freezing. We hypothesized that the second ejaculate of the season would have superior semen quality after cooling and freezing to the first ejaculate. Additionally, we hypothesized that semen processing via cushion-centrifugation would improve its quality.

## 2. Materials and Methods

The present study was performed at the Veterinary Teaching Hospital of the University of Illinois Urbana-Champaign, IL, USA. The Institutional Animal Care Unit Committee approved all the procedures in the present experiment under protocol #19134.

### 2.1. Stallions

A total of 20 mature stallions (11.7 ± 1.5 years old, range 4–23 years old) were enrolled in the study (Table 1). The criteria for enrollment included being sexually mature (≥4 years old), having two testes present in the scrotum, the absence of apparent abnormalities of the reproductive tract, and being breeding inactive for at least four months before presentation to the Equine Theriogenology Service at the Veterinary Teaching Hospital of the University of Illinois Urbana-Champaign during the 2022 reproductive season in the Northern Hemisphere. The stallions enrolled in the study were client-owned breeding animals presented for semen collection, evaluation, shipping, and freezing, or for washout semen collections, at the onset of the reproductive season (Table 1).

### 2.2. Study Design, Semen Cooling, Freezing, and Post-Thaw Thermal Longevity Test

Two ejaculates (n = 40) were collected 1 h apart from each stallion. The semen collections were performed using a dummy mount in the presence of a teaser mare in good standing estrus in a Missouri artificial vagina (Nasco, Fort Atkinson, WI, USA) coupled with an in-line filter (Har-Vet, Elmwood, WI, USA).

After each semen collection, the gel-free volume, sperm concentration, total number of sperm, and sperm morphology were determined. An aliquot of each ejaculate was extended in a commercially available phosphocaseinate extender (INRA96^®^, IMV Technologies USA, Brooklyn Park, MN, USA) at 50 million sperm/mL and then placed in a passive cooling device (Botuflex^®^, Botupharma USA, Phoenix, AZ, USA) for 48 h at 5 °C. Another aliquot of each ejaculate was also extended at 50 million sperm/mL on the same extender and then cushion-centrifuged (1000× *g* × 20 min) as previously described [7,8,9]. The supernatant was discarded, and the pellet was resuspended with the same extender at 50 million sperm/mL and cooled for 48 h [9]. The remainder of each ejaculate was extended in a commercially available phosphocaseinate extender (INRA 96, Maple Grove, MN, USA) and cushion-centrifuged. The pellet was resuspended in a commercially available egg-yolk-based freezing extender (Botucrio^®^ Botupharma USA, Phoenix, AZ, USA) at 200 million sperm/mL, frozen over liquid nitrogen vapor, and then plunged into liquid nitrogen [6]. 

After at least two weeks of freezing, two straws from each ejaculate were thawed at 37 °C for 60 s. The semen was pooled in a 15 mL conical tube and then assessed for the motility parameters, sperm plasma membrane integrity, and sperm with high mitochondrial potential, as described below. The thawed semen was further incubated at 37 °C, and the motility parameters were evaluated every 30 min up to 240 min for the completion of a thermal longevity test [15,16,17]. 

The stallions were logically classified based on the variation or lack of variation between the first and second ejaculates for the motility parameters as follows: *unchanged*, no variation between the first and second ejaculates, when the variation was ≤10%; *increased*, if the second ejaculate had an improvement of >10% in the motility parameters when compared with the first ejaculate; or *decreased* if the second ejaculate had a reduction of >10% in comparison with the first ejaculate. This stallion classification was used for the analyses of all the endpoints in addition to the non-grouped data, and it was intended to provide some clinical references for practitioners collecting and processing semen from breeding breeding stallions. 

### 2.3. Semen Volume, Sperm Concentration, Total Number of Sperm, and Morphology

The gel-free semen was placed on a digital scale, and the volume was recorded. The sperm concentration was assessed using an automated cell counter (NucleoCounter SP100™, Chemometec, Alleród, Denmark) following the manufacturer’s recommendations. Briefly, 50 µL of semen was diluted in 5 mL of reagent S100 (Chemometec, Alleród, Denmark), and the SP1 cassette was loaded into the instrument for the assessment. The total number of sperm was obtained by multiplying the sperm concentration by the gel-free semen volume. 

For the sperm morphology evaluation, an aliquot (50 µL) of raw semen was mixed with 1 mL of buffered formalin 10%. The same theriogenologist assessed a wet-mount preparation from each ejaculate. On each wet mount, 100 sperm were counted and classified based on the absence of morphological abnormalities or the presence and type of morphological abnormality. The percentages of morphologically normal sperm were used for the comparisons across stallions and ejaculates alongside the different morphologic sperm abnormalities.

### 2.4. Sperm Motility Assessment

The sperm motility parameters were assessed pre-(0 h), 24 h, and 48 h post-cooling as well as before and after freezing [9,10]. The motility parameters were assessed with a computer-assisted sperm analyzer using the default settings recommended by the manufacturer (SpermVision, Minitube of America, Verona, WI, USA) for equine sperm. The CASA’s preset values were a static cell area of 14–80 µm^2^; a straightness threshold for progressive motility of 90%; an average path velocity threshold for a static cell of <9.5 µm/s; a cell intensity of 10^6^; and a light-emitting diode illumination intensity of 1800–2550. Each sample (10 µL) was incubated for 10 min at 37 °C before each evaluation. Up to 1000 sperm or 15 fields randomly selected were analyzed in each sample. The motility parameters assessed included the total percentage of sperm motility (TM, %), progressive sperm motility (PM, %), average path velocity (VAP, μm/s), curvilinear velocity (VCL, μm/s), and straight-line velocity (VSL, μm/s).

### 2.5. Sperm Plasma Membrane Integrity and Mitochondrial Membrane Potential

The sperm membrane integrity and mitochondrial membrane potential were assessed pre-(0 h), 24 h, and 48 h post-cooling and after freezing and thawing [9]. The evaluation of the plasma membrane integrity and mitochondrial membrane potential was conducted using a spectral flow cytometer, as previously described [18] and as extensively used in the laboratory of the investigators of the present study [9,19,20,21]. Briefly, the staining solution of Zombie Green dye (#423112 BioLegend, San Diego, CA, USA) was freshly prepared with 100 μL of DMSO added to each vial of dye. Similarly, the MitoTracker Deep Red FM (M22426, Molecular Probes, Eugene, OR, USA) stock solution was prepared by adding DMSO to create a 10 μM working solution. The stock solution was aliquoted and frozen at −20 °C until it was used.

**Figure 1:**
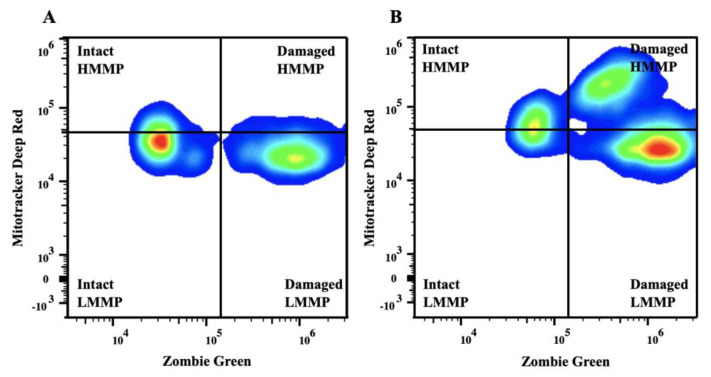
Representative flow cytometry plots of semen collected from a breeding inactive stallion before (**A**) and after (**B**) freezing. Sperm were stained with Zombie Green and MitoTracker Deep Red. The upper quadrants represent intact (**left**) and damaged (**right**) sperm with high mitochondrial membrane potential. The lower quadrants represent intact (**left**) and damaged (**right**) sperm with low mitochondrial membrane potential. Abbreviations: Intact, sperm with intact plasma membrane; HMMP, intact sperm with high mitochondrial membrane potential; LMMP, intact sperm with low mitochondrial membrane potential.

One milliliter containing 50 million sperm/mL was centrifuged (600× *g* × 10 min) and then resuspended in PBS at a sperm concentration of 3–5 million sperm/mL. Subsequently, a 100 μL aliquot of this solution was stained with both dyes (1 μL of Zombie Green and 1 μL of MitoTracker Deep Red). After mixing, the sample was incubated for 30 min at room temperature in the dark. The incubation was followed by centrifugation (400× *g* × 5 min). The supernatant was discarded, and each pellet was fixed with 250 μL of 2% buffered formalin and stored in the dark until the flow cytometric evaluation. Before the flow cytometric analysis, the samples were washed with 1 mL of PBS, centrifuged at 400× *g* × 5 min, and resuspended in PBS (250 μL). The analyses of the stained samples were conducted using a full-spectrum detector-based (filter-less) Cytek Aurora Flow Cytometer (Cytek Biosciences Inc., Fremont, CA, USA). The analysis was completed when at least 10,000 fluorescent gated events were recorded. The Zombie Green was excited and detected with a 488 nm fluorescence detector, whereas the MitoTracker Deep Red was excited with a 644/665 nm detector. Unstained and single-stained controls were used to unmix the signals. As previously described, four subpopulations of sperm were identified [12]. The populations of sperm with intact (low Zombie Green signal) or damaged (high Zombie Green signal) plasma membrane were subdivided into low or high mitochondrial membrane potential based on the intensity of the signal given by the MitoTracker Deep Red staining. Any debris was manually excluded based on the minimal emitted fluorescence. The data from the flow cytometer were exported and analyzed with FlowJo (FlowJo v.10 Software, Ashland, OR, USA). The percentage of sperm plasma membrane integrity and the percentage of sperm plasma membrane integrity with high mitochondrial membrane potential were used for the comparisons across the groups, and a representative figure is depicted below (Figure 1).

### 2.6. Statistical Analyses

The normal distribution of the results was verified by a Shapiro–Wilk test and residual plots. The gel-free semen volume, sperm concentration, total number of sperm, sperm morphology, motility parameters, plasma membrane integrity, and sperm with plasma membrane integrity with high mitochondrial membrane potential were compared with a paired t-test between the first and second ejaculates. The cooling, freezing, and post-thaw sperm parameters were also analyzed with a linear mixed model. The ejaculate (first vs. second), the processing (cushion-centrifugation vs. non-centrifugation), the freezing, and the time (cooling and post-thaw thermal longevity test) were accounted for as fixed effects. By contrast, the stallion was considered as a random effect. The statistical analyses were also performed with the stallions grouped as *unchanged*, *increased*, and *decreased* regarding the variation in the motility parameters between the first and second ejaculates. A two-way analysis of variances was performed to determine the effect of the grouping and the time or freezing on the motility parameters. Significance was set at *p* < 0.05. The data were presented as the mean ± SEM. The 95% confidence interval were displayed for the gel-free semen volume, sperm concentration, total number of sperm, sperm morphology, and motility parameters. 

## 3. Results

### 3.1. Raw Semen Parameters of the First and Second Ejaculates of the Reproductive Season

The second ejaculate had a lower gel-free semen volume (85.2 ± 8.5 mL vs. 65.2 ± 4.8 mL) (*p* = 0.026, ~76%) (Table 2). The sperm concentration was greater in the first ejaculate than in the second ejaculate (*p* < 0.001) (Table 2). Overall, the second ejaculate yielded 56% of the total sperm of the first ejaculate, and five stallions had a second ejaculate with >50% of the total sperm obtained in the first ejaculate. Five stallions had a second ejaculate with 40 to 50% of the total sperm obtained in the first ejaculate, four stallions between 30 and 40%, and three stallions between 20 and 30%. Three stallions had higher total sperm in the second ejaculate than the first. 

All the stallions had at least 1 billion total sperm in the second ejaculate. Based on the motility parameters, sixteen stallions were classified as *unchanged* (i.e., ≤10% change), three stallions as *increased* (i.e., improvement > 10%) group, and one stallion as *decreased* (i.e., reduction > 10%). There was no difference in the gel-free volume, sperm concentration, and total sperm across the groups (*p* > 0.05) (Table 3).

The sperm morphology did not vary between the ejaculates (*p* > 0.05) (Table 4). The first ejaculate tended to have a greater percentage of sperm with proximal protoplasmatic droplets (*p* = 0.0816) (Table 4). There was no difference in the percentage of morphologically normal sperm or other sperm abnormalities across the groups (*unchanged*, *increased*, and *decreased*) (*p* > 0.05) (Table 5). There was no difference in the motility parameters, sperm plasma membrane integrity, and high mitochondrial membrane potential between the first and second ejaculates when the stallions were not categorized into groups (*p* > 0.05) (Table 6). There was no difference in the total motility, progressive motility, VCL, VSL, and VAP between the groups (*p* > 0.05) (Table 7).

### 3.2. Semen Cooling

The total and progressive sperm motilities varied over time (*p* < 0.001) and by the cushion-centrifugation (*p* < 0.001) but not by the ejaculate (*p* = 0.80). There was no interaction for the total and progressive motilities between the cushion-centrifugation and the ejaculates (*p* = 0.2331 and *p* = 0.995, respectively) (Table 8). The non-centrifuged samples had a more marked reduction in the total and progressive motility over time than the centrifuged semen (*p* < 0.05) (Table 8). The values of the VCL, VSL, and VAP decreased over time (*p* < 0.001) but did not differ between the ejaculates (*p* > 0.05) or after the cushion-centrifugation (*p* > 0.05) (Table 8). There was no interaction between the ejaculate and centrifugation (VCL, *p* = 0.574; VSL, *p* = 0.3751; and VAP, *p* = 3473) or between the time and the ejaculate (VCL, *p* = 0.7329; VSL, *p* = 0.5708; and VAP, *p* = 0.5579) during cooling (Table 8). The values of the VCL, VSL, and VAP decreased between time 0 and 48 h of cooling regardless of centrifugation and the ejaculate (*p* < 0.001) (Table 8).

The cushion-centrifuged samples had a greater percentage of sperm with plasma membrane integrity than the non-centrifuged semen (*p* < 0.001), although it did not influence the mitochondrial membrane potential (*p* = 0.739) (Table 9). There was no effect of the time or of the ejaculate on the plasma membrane integrity and mitochondrial membrane potential during cooling (*p* > 0.05). There were no interactions between the cushion-centrifugation, time, or ejaculate (*p* > 0.05) (Table 9).

There were effects from the groups (*p* < 0.001) and the time (*p* < 0.001), but not from their interactions (*p* > 0.05), on the total motility and progressive motility during cooling. The *unchanged* and *decreased* groups had greater total and progressive sperm motilities than the *increased* group (*p* < 0.001). Conversely, the groups did not affect the VAP (*p* = 0.2576), VCL (*p* = 0.093), or VSL (*p* = 0.3151) (Table 10 and Table 11). 

### 3.3. Post-Thaw Semen Parameters and Thermal Longevity Test

**Figure 2:**
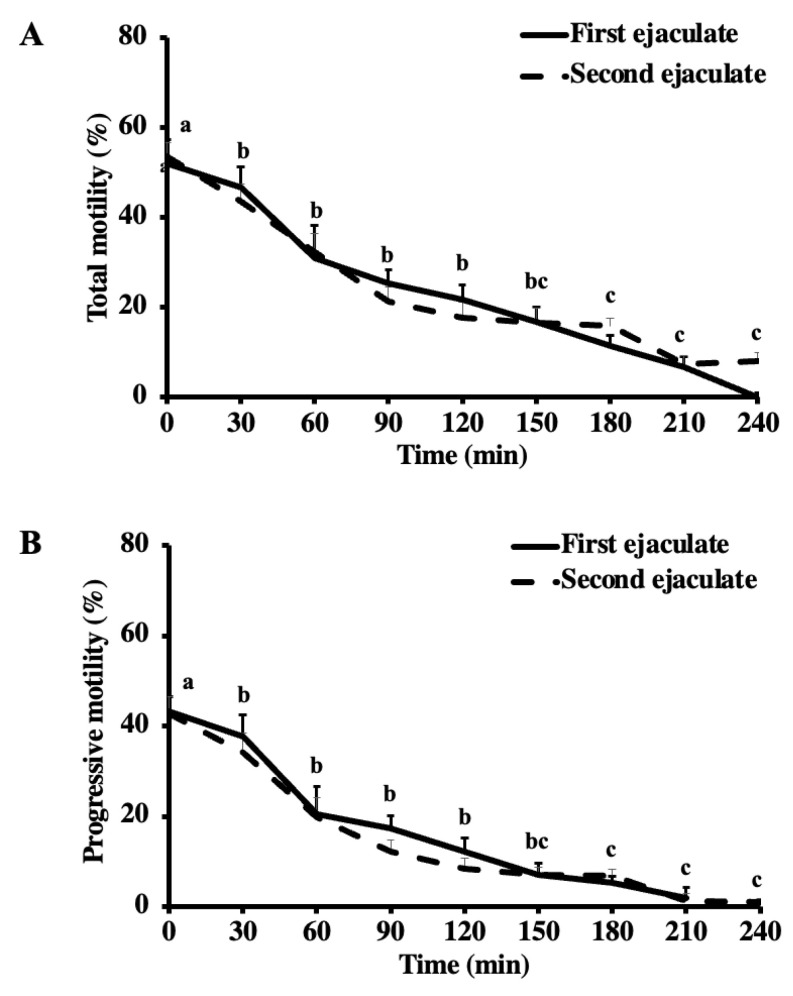
Total (**A**) and progressive (**B**) motility of the first and second ejaculates from breeding inactive stallions (n = 20) during the thermal longevity test. The frozen-thawed semen was incubated at 37 °C for 240 min. Different superscripts (^abc^) denote a difference between columns (*p* < 0.05).

**Figure 3:**
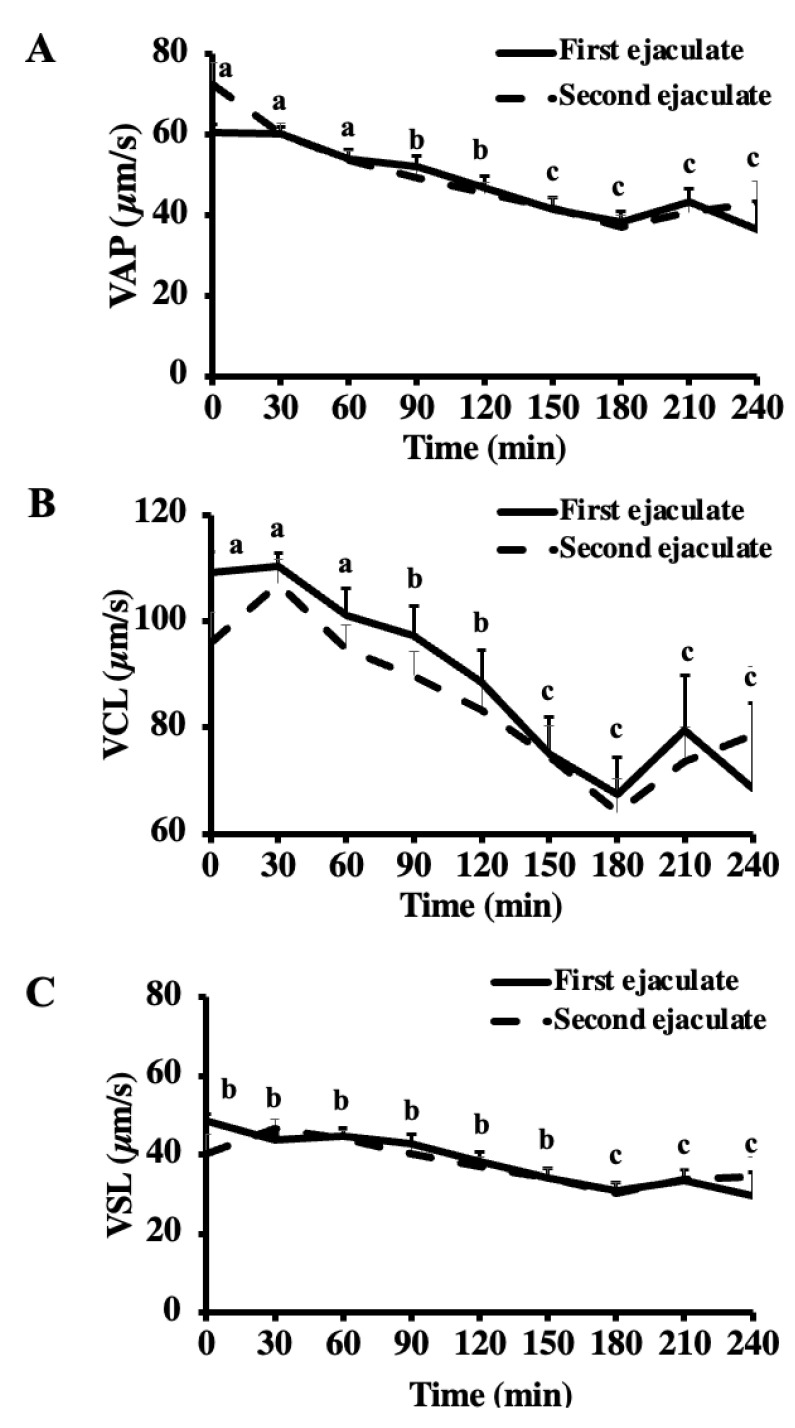
Velocity parameters ((**A**) VAP; (**B**) VCL; (**C**) VSL) of the first and second ejaculates collected from breeding inactive stallions (n = 20). The frozen-thawed semen was incubated at 37 °C for 240 min. Different superscripts (^abc^) denote a difference between columns (*p* < 0.05).

The total and progressive motilities decreased from the pre-freezing to the post-thaw (*p* < 0.001) but did not vary between the ejaculates (*p* = 0.889 and *p* = 0.276, respectively) (Table 12). During the thermal longevity test, there was a reduction in the total and progressive motility by 30 and 120 min of the thermal longevity test (*p* < 0.05) (Figure 2). The sperm velocity parameters, VCL, VSL, and VAP, decreased by the post-thaw (*p* < 0.05) and during the thermal longevity test (*p* < 0.05), although there was no effect of the ejaculate and no interactions (*p* > 0.05) (Table 12, Figure 3). The values of the VCL and VAP decreased after 150 min of the thermal longevity test (*p* < 0.05). The VSL decreased just after 180 min of the test (*p* < 0.05). The percentage of sperm with intact plasma membrane decreased from the pre-freezing to the post-thaw (*p* < 0.001), but it did not vary with the ejaculate (*p* = 0.936) (Table 12). The percentage of sperm with high mitochondrial membrane potential did not vary post-thaw (*p* = 0.289), with the ejaculate (*p* = 0.396), or with their interactions (*p* = 0.612) (Table 12). 

**Figure 4:**
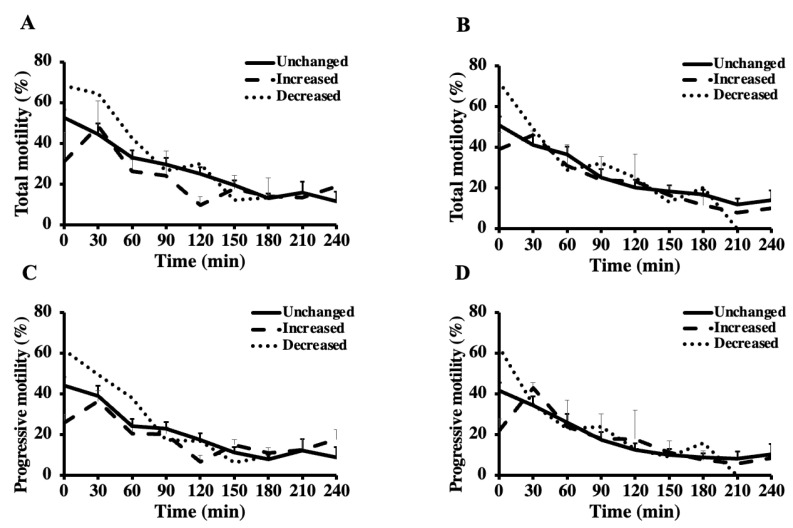
Total (**A**,**B**) and progressive (**C**,**D**) motility of the first (**A**,**C**) and second (**B**,**D**) ejaculates from breeding inactive stallions (n = 20) during a thermal longevity test. The frozen-thawed semen was incubated at 37 °C for 240 min. Based on the motility parameters between the first and second ejaculates, sixteen stallions were classified as *unchanged* (i.e., ≤10% change), three stallions as *increased* (i.e., improvement > 10%) group, and one stallion as *decreased* (i.e., reduction > 10%).

When the stallions were compiled into three groups, the total and progressive motilities were influenced by the freezing (*p* < 0.001) and the groups (*p* < 0.001) but not by their interactions (*p* > 0.05) (Table 13, Figure 4). The *unchanged* and *decreased* groups had greater motility than the *increased* group (*p* < 0.05) (Table 13). The VAP (*p* = 0.2586), VCL (*p* = 0.069), and VSL (*p* = 0.25) were not influenced by the groups, although they were influenced by the freezing (*p* < 0.001) (Table 13). During the thermal longevity test, the total and progressive motility, VAP, VCL, and VSL were not influenced by the groups, ejaculate, or the interaction between them (*p* > 0.05), although they were influenced by the time (Figure 4). Both the total and progressive motility, VAP, VCL, and VSL decreased over time in every group (*p* < 0.05) (Figure 4 and Table 13).

## 4. Discussion

The present study aimed to assess the semen parameters and the cooling and freezing abilities of breeding inactive stallions’ semen collected 1 h apart during the physiological reproductive season in the Northern Hemisphere. The motility parameters changed between the first and second ejaculates. Here, 80% of the stallions presented minimal variations between the ejaculates (≤10%, *unchanged*), whereas 15% improved (*increased*) and 5% displayed a reduction (*reduced*). The pattern of variation between first and second ejaculates appeared to influence how the stallions responded to cooling and freezing. Interestingly, the increase in the motility parameters between the first and second ejaculates was associated with a reduction in the sperm motility parameters during cooling and post-thaw; however, due to the limited number of animals in the groups changing, we were unable to find significant differences. In addition, performing a second semen collection may not necessarily mean that the second ejaculate should be used for further processing instead of the first. However, a limitation of the study is that fertility was not evaluated, while a second limitation is that a reduced number of stallions classified as *increased* were enrolled. Yet, these findings suggest that stallions with variations should be further investigated regarding their cooling and freezing abilities and, ultimately, their fertility.

In clinical practice, once a breeding inactive stallion is hastily presented for semen collection and shipping, most practitioners typically procure and evaluate the first ejaculate, extend the semen in a cooling extender at an equal volume, and assess the sperm parameters. Based on the sperm motility parameters of the first ejaculate or not, some practitioners and farm managers do not collect a second ejaculate and instead cool and ship out the first ejaculate of the season regardless of the semen quality. However, others collect a second ejaculate, at least 1 h apart, if the stallion has adequate libido and physical capabilities (e.g., free from major orthopedic pain or neurological problems) to collect semen in a short interval. While a second ejaculate is attempted, the semen collected from the first ejaculate is maintained in an incubator at 37 °C or room temperature. If the second ejaculate has better quality than the first ejaculate, practitioners process the second ejaculate for on-farm use, cooling-shipping, or freezing. If both ejaculates have identical marginal to poor quality, either ejaculate is processed further, or both ejaculates are discarded and additional ejaculates are collected on the same day or following days. The latter is particularly needed for stallions classified as sperm accumulators [22]. The present study was carried out under practical conditions reproducing a common clinical scenario faced by many practitioners in North America. The current findings appear to support the practice of extending and procuring a second ejaculate and potentially processing the first, the second, or both ejaculates. In the authors’ clinical practice, all breeding inactive stallions hastily presented for semen collection and shipping have semen extended and cushion-centrifuged. The study findings demonstrated that this approach prevents a reduction in sperm motility during cooling rather than shipping non-centrifuged semen. The sperm recovery during cushion-centrifugation was not evaluated herein, but at least 95% recovery should be expected when correctly applying this technique [7]. In addition, sperm recovery should not be a concern for the first and second ejaculates of the season, as breeding inactive stallions typically ejaculate two- to five-fold greater total sperm [3,4,23]. It was not different in the present study as the stallions in this study well surpassed the expected sperm counts of light breed stallions [23,24].

The tendency for the first ejaculate to have a greater percentage of sperm cells with protoplasmatic droplets could be due to the high sperm concentration in the first ejaculate. However, many authors consider proximal droplets to be non-important sperm morphologic defects [25,26,27], and it has also been reported that thoroughbred stallions under natural mating with a higher percentage of this morphologic defect have higher fertility [27]. It remains to be determined how stallions under artificial insemination are affected by the proximal droplets. 

Semen collection from breeding inactive stallions is also performed at 1 h apart for breeding soundness evaluation and semen cooling and freezing tests [28,29]. However, scant evidence supports or refutes the use of the first ejaculate of the reproductive season for semen cooling and freezing testing. Most stallions in the present study were classified as fertile based on their past reproductive history. It remains to be determined if the results would have been different with more stallions that had poor semen quality enrolled in the study. Sperm accumulators would change the results drastically because this type of stallion usually has all immotile sperm when breeding inactive breeding [22]; however, stallions with marginal semen quality likely would have had different results. The effects of the season on stallion semen cooling and freezing are somewhat controversial, and it has been suggested that winter potentially affects the semen [30]. A study showed that the post-thawed motility was greater in semen frozen during the winter than that frozen during the spring [31]. However, more recent studies supported the idea that the optimal time for semen freezing is the fall [32,33,34]. While the present study was performed in the early spring, with a spermatogenesis of ~60d [23,24], the sperm harvested was produced during the winter months. As the effects of breeding inactivity were not compared across seasons, it remains to be determined if and how the season affects the semen quality in breeding inactive stallions. 

The present study supports the notion that breeding inactive stallions have satisfactory semen quality after cooling and freezing and that cushion-centrifugation enhances the motility and viability after cooling. Practitioners frequently perform semen centrifugation after collecting breeding inactive stallions’ semen; thus, our findings support, at least in vitro, the use of this procedure in clinical practice. Semen centrifugation is routinely used in clinical practice to remove the seminal plasma and the concentration of sperm before cooling-shipping, freezing, or deep-horn insemination after cooling [9]. Removing the seminal plasma increases the sperm motility parameters and the longevity of semen cooled and collected from breeding active stallions [7,8,9]. Previously, traditional/conventional centrifugation was widely used over cushion-centrifugation; however, in recent years, cushion-centrifugation has become a new standard in equine practice, as it allows for sperm recovery greater than 95% with no detrimental effects on the sperm quality [7]. In the United States, semen centrifugation is not routinely applied before semen cooling and shipping unless the stallion has a low sperm concentration (<100 million sperm/mL), a history of poor semen quality during cooling (i.e., a drastic decline in sperm motility after cooling), or urine contamination [10,11]. Conversely, in other countries, centrifugation is routinely used in almost all stallions before semen cooling and shipping. Furthermore, in many equine breeding facilities, centrifugation is also used to process semen from breeding inactive stallions before cooling and shipping. The present study’s findings confirmed that semen centrifugation prevents the loss of sperm motility during the cooling of semen from breeding inactive stallions. In addition, one finding of the study was that there was a mismatch between the motility parameters and plasma membrane. This discrepancy could be due to the techniques used to evaluate these parameters; for instance, if a non-fixative method was used for the flow cytometry analyses, different results could have been found.

## 5. Conclusions

In conclusion, the first and second ejaculates of the season collected 1 h apart varied in quantity but not in quality. Cushion-centrifugation prevented the reduction of the sperm motility during cooling. The post-thaw semen quality did not vary between the first and second ejaculates. Although the fertility was not tested, the results suggest that there is minimal variation between the first and second ejaculates from breeding inactive stallions and that both are suitable for further processing for cooling and freezing. 

## Figures and Tables

**Table 1:** Signalment, history, and reason for the presentation of the 20 breeding inactive stallions.

Stallion	Breed	Age (Years)	Pertinent Breeding History	Reason for Presentation
1	Quarter Horse	9	Low sperm count, seminal plasma toxicity	Washout collections
2	Quarter Horse	13	Fertile	Washout collections
3	Quarter Horse	11	Fertile	Washout collections
4	Quarter Horse	23	Used for live cover	Semen freezing
5	Paint Horse	5	Maiden	Training to collect semen
6	Quarter Horse	15	Fertile	Washout collections
7	Standardbred	11	Fertile	Washout collections
8	Standardbred	22	Fertile	Washout collections
9	Paint Horse	14	Low sperm count, seminal plasma toxicity	Washout collections
10	Quarter Horse	7	Fertile	
11	Standardbred	11	Sperm accumulator, seminal plasma toxicity	Washout collections
12	Standardbred	15	Fertile	Washout collections
13	Standardbred	4	Low fertility and inconsistent breeder	Washout collections
14	Standardbred	6	Fertile	Washout collections
15	Standardbred	9	Fertile	Washout collections
16	Arabian	21	Fertile	Washout collections
17	Arabian	19	Fertile	Washout collections
18	Quarter Horse	13	Fertile	Washout collections
19	Andalusian	8	Unknown	Semen shipping
20	Standardbred	14	Fertile	Semen shipping

**Table 2:** Gel-free semen volume, sperm concentration, and total number of sperm of raw semen from the first (n = 20) and second ejaculates (n = 20) of breeding inactive stallions (n = 20).

	First Ejaculate		Second Ejaculate	
	Mean ± SEM	CI-95%	Mean ± SEM	CI-95%
Gel-free volume (mL)	83.5 ± 8.1 ^a^	66.5–100.5	68.8 ± 5.0 ^b^	58.3–79.4
Sperm concentration (million/mL)	245 ± 33.8 ^a^	174.4–316.1	146.0 ± 28.6 ^b^	86.1–205.7
Total number of sperm (billion)	17.8 ± 2.5 ^a^	12.3–23.1	10.3 ± 3.9 ^b^	4.0–16.0

Different superscripts within column denote differences within the same endpoint (*p* < 0.05).

**Table 3:** Gel-free semen volume, sperm concentration, and total number of sperm of raw semen from the first and second ejaculates of breeding inactive stallions (n = 20). Based on the motility parameters between the first and second ejaculates, sixteen stallions were classified as *unchanged* (i.e., ≤10% change), three stallions as *increased* (i.e., improvement > 10%) group, and one stallion as *decreased* (i.e., reduction > 10%).

	*Unchanged* (n = 16)		*Increased* (n = 3)		*Decreased* (n = 1)	
	First Ejaculate	Second Ejaculate	First Ejaculate	Second Ejaculate	First Ejaculate	Second Ejaculate
	Mean ± SEM	Mean ± SEM	Mean ± SEM	Mean ± SEM		
Gel-free volume (mL)	79.3 ± 7.8	67.5 ± 5.3	124.0 ± 19.9	62.0 ± 12.5	30	37
Sperm concentration (million/mL)	255.7 ± 40	150.7 ± 35	162.0 ± 31	117.0 ± 28	328	154
Total number of sperm (billion)	17.6 ± 3.0	11.0 ± 3.6	21.3 ± 6.4	7.9 ± 3.4	9.8	5.6

**Table 4:** Sperm morphology of the first and second ejaculates of the season collected from 20 breeding inactive stallions.

	First Ejaculate		Second Ejaculate	
	Mean ± SEM	CI-95%	Mean ± SEM	CI-95%
Normal (%)	62.7 ± 4.5	52.9–72.5	67.6 ± 4.6	57.9–77.2
Proximal droplet (%)	13.4 ± 4.2	5.4–22.7	12.9 ± 3.8	3.9–18.6
Distal droplet (%)	12.3 ± 2.5	1.8–13.8	5.0 ± 1.5	0.9–7.7
Tailless (%)	2.8 ± 0.6	0.7–3.4	1.3 ± 0.4	0.3–2.2
Simple bent (%)	5.5 ± 1.4	2.8–9.5	6.7 ± 1.4	3.1–9.2
Strongly folded (%)	1.7 ± 0.4	1.1–3.5	2.1 ± 0.6	1.2–5.4
Midpiece defects (%)	0.8 ± 0.4	1–1.8	0.9 ± 0.4	1–1.6

**Table 5:** Sperm morphology of the first and second ejaculates of the season collected from 20 breeding inactive stallions. Based on the motility parameters between the first and second ejaculates, sixteen stallions were classified as *unchanged* (i.e., ≤10% change), three stallions as *increased* (i.e., improvement > 10%), and one stallion as *decreased* (i.e., reduction > 10%).

	*Unchanged* (n = 16)		*Increased* (n = 3)		*Decreased* (n = 1)	
	First Ejaculate	Second Ejaculate	First Ejaculate	Second Ejaculate	First Ejaculate	Second Ejaculate
	Mean ± SEM	Mean ± SEM	Mean ± SEM	Mean ± SEM		
Normal (%)	63.4 ± 5.4	69.8 ± 4.8	58.3 ±13.3	52.0 ± 3.0	67	37
Proximal droplet (%)	14.7 ± 4.0	10.4 ± 3.1	5.6 ± 3.3	9.5 ± 4.5	22	154
Distal droplet (%)	10.4 ± 3.5	8.3 ± 3.5	22.3 ± 11.6	29.5 ± 9.5	6	5.6
Tailless (%)	3.4 ± 1.1	2.2 ± 3.5	1.0 ± 0.6	2.5 ± 1.5	1	2
Simple bent (%)	5.6 ± 1.5	5.5 ± 1.3	7.0 ± 3.2	5.0 ± 5.0	0	0
Strongly folded (%)	1.9 ± 0.5	2.7 ± 0.9	3.7 ± 2.3	8.0 ± 3.0	3	0
Midpiece defects (%)	0.6 ± 0.3	0.9 ± 0.4	2.0 ± 1.5	0.5 ± 0.5	0	3

**Table 6:** Qualitative comparison of the first and second ejaculates collected from breeding inactive stallions (n = 20).

	First Ejaculate		Second Ejaculate	
	Mean ± SEM	CI-95%	Mean ± SEM	CI-95%
TM (%)	65.4 ± 5.6	53.5–77.2	67.8 ± 3.3	60.7–74.9
PM (%)	58.9 ± 5.4	47.4–70.3	61.7 ± 3.2	54.8–68.6
VCL (μm/s)	147.8 ± 4.0	140.4–156.8	151.4 ± 4.5	141.3–159.5
VSL (μm/s)	69.7 ± 3.5	62.3–76.2	75.3 ± 3.5	73.9–3.5
VAP (μm/s)	87.2 ± 3.9	79.3–94.7	91.9 ± 3.5	83.0–98.1
PMI (%)	68.0 ± 6.1	53.7–82.3	66.5 ± 5.6	53.9–79.1
HMMP (%)	40.5 ± 2.5	34.9–46.1	41.3 ± 4.0	32.1–50.5

Abbreviations: TM, total sperm motility; PM, progressive sperm motility; VCL, curvilinear velocity; VSL, straight-line velocity; VAP, average path velocity; PMI, plasma membrane integrity; HMMP, intact sperm with high mitochondrial membrane potential.

**Table 7:** Qualitative comparison of the first and second ejaculates collected from breeding inactive stallions (n = 20). Based on the motility parameters between the first and second ejaculates, sixteen stallions were classified as *unchanged* (i.e., ≤10% change), three stallions as *increased* (i.e., improvement >10%), and one stallion as *decreased* (i.e., reduction >10%).

	*Unchanged* (n = 16)		*Increased* (n = 3)		*Decreased* (n = 1)	
	First Ejaculate	Second Ejaculate	First Ejaculate	Second Ejaculate	First Ejaculate	Second Ejaculate
	Mean ± SEM	Mean ± SEM	Mean ± SEM	Mean ± SEM		
TM (%)	71.3 ± 5.3	71.0 ± 4.3	32.2 ± 11.1	59.0 ± 5.9	86.6	74.3
PM (%)	64.0 ± 5.0	64.0 ± 4.2	26.4 ± 9.5	54.6 ± 6.4	82.2	69.0
VCL (μm/s)	145.7 ± 4.7	150.7 ± 5.2	143.9 ± 8.3	140.6 ± 0.8	164.4	162.9
VSL (μm/s)	67.7 ± 4.4	74.4 ± 4.3	66.2 ± 10.8	72.1 ± 9.5	81.1	81.4
VAP (μm/s)	85.4 ± 4.5	91.5 ± 4.1	83.7 ± 13.9	86.4 ± 11.7	100.0	97.4

**Table 8:** Motility parameters of the first and second ejaculates of the season collected from breeding inactive stallions (n = 20) after 0, 24, and 48 h of cooling.

		Non-Centrifuged		Cushion-Centrifuged	
	Time (h)	First Ejaculate	Second Ejaculate	First Ejaculate	Second Ejaculate
TM (%)	0	65.4 ± 5.6 ^a^	67.8 ± 3.3 ^a^	63.0 ± 5.1 ^a^	62.7 ± 3.9 ^a^
	24	50.3 ± 7.1 ^b^	48.1 ± 5.6 ^b^	56.7 ± 5.5 ^b^	59.8 ± 5.0 ^b^
	48	41.4 ± 7.0 ^b^	33.4 ± 4.3 ^b^	51.4 ± 5.3 ^b^	59.9 ± 5.4 ^b^
PM (%)	0	59.4 ± 5.6 ^a^	62.8 ± 3.6 ^a^	55.4 ± 5.0 ^a^	53.6 ± 4.6 ^a^
	24	43.3 ± 7.3 ^b^	39.5 ± 5.3 ^b^	50.4 ± 5.3 ^b^	52.3 ± 4.9 ^b^
	48	35.5 ± 7.1 ^b^	26.4 ± 4.0 ^b^	44.6 ± 5.3 ^b^	52.7 ± 5.7 ^b^
VAP (μm/s)	0	87.2 ± 3.9 ^a^	91.9 ± 3.5 ^a^	72.8 ± 5.5 ^a^	90.3 ± 4.7 ^a^
	24	70.9 ± 5.2 ^ab^	76.0 ± 6.5 ^ab^	72.5 ± 4.5 ^ab^	72.5 ± 4.2 ^ab^
	48	62.2 ± 6.9 ^b^	71.1 ± 3.5 ^b^	68.1 ± 4.1 ^b^	70.4 ± 3.7 ^b^
VCL (μm/s)	0	147.8 ± 4.0 ^a^	151.4 ± 4.5 ^a^	135.0 ± 8.8 ^a^	140.6 ± 7.6 ^a^
	24	122.9 ± 8.3 ^ab^	132.6 ± 9.9 ^ab^	132.1 ± 5.8 ^ab^	127.7 ± 6.2 ^ab^
	48	114.7 ± 12.8 ^b^	136.7 ± 6.2 ^b^	125.6 ± 4.5 ^b^	122.7 ± 4.7 ^b^
VSL (μm/s)	0	69.7 ± 3.5 ^a^	75.3 ± 3.5 ^a^	56.7 ± 4.2 ^a^	61.7 ± 3.9 ^a^
	24	58.6 ± 4.0 ^ab^	62.5 ± 5.9 ^ab^	59.6 ± 4.1 ^ab^	59.4 ± 3.6 ^ab^
	48	49.5 ± 5.3 ^b^	53.8 ± 2.5 ^b^	56.1 ± 3.5 ^b^	58.5 ± 3.4 ^b^

Abbreviations: TM, total motility; PM, progressive motility; VAP, average path velocity; VCL, curvilinear velocity; VSL, straight-line velocity. Different superscripts (^ab^) denote differences within the column (*p* < 0.05).

**Table 9:** Percentage of sperm with an intact membrane (PMI) and high mitochondrial membrane potential (HMMP) from the first and second ejaculates of the season collected from breeding inactive stallions (n = 20) after 0, 24, and 48 h of cooling.

		Non-Centrifuged		Cushion-Centrifuged	
	Time (h)	First Ejaculate	Second Ejaculate	First Ejaculate	Second Ejaculate
PMI (%)	0	66.3 ± 5.6	66.3 ± 6.2	71.5 ± 3.7	69.8 ± 5.0
	24	53.9 ± 6.4	53.5 ± 9.9	66.9 ± 5.5	63.5 ± 6.5
	48	53.6 ± 8.9	52.0 ± 10.7	67.7 ± 5.6	69.9 ± 7.2
HMMP (%)	0	38.8 ± 2.5	41.3 ± 4.0	42.0 ± 2.9	39.4 ± 3.8
	24	40.6 ± 4.3	36.0 ± 3.0	45.7 ± 9.9	40.5 ± 6.7
	48	50.7 ± 10.0	46.0 ± 4.3	42.5 ± 3.1	38.7 ± 6.3

Abbreviations: PMI, plasma membrane integrity; HMMP, intact sperm with high mitochondrial membrane potential.

**Table 10:** Motility parameters of the first and second ejaculates of the season collected from breeding inactive stallions (n = 20) after 0, 24, and 48 h of cooling. Based on the motility parameters between the first and second ejaculates, sixteen stallions were classified as *unchanged* (i.e., ≤10% change), three stallions as *increased* (i.e., improvement > 10%) group, and one stallion as *decreased* (i.e., reduction > 10%).

		*Unchanged* (n = 16)		*Increased* (n = 3)		*Decreased* (n = 1)	
	Time (h)	First Ejaculate	Second Ejaculate	First Ejaculate	Second Ejaculate	First Ejaculate	Second Ejaculate
TM (%)	0	71.3 ± 5.3	71.0 ± 4.3	32.3 ± 11.1	59.0 ± 5.9	86.6	74.3
	24	53.9 ± 6.9	51.1 ± 5.2	23.2 ± 13.5	27.2 ± 11.5	88.2	77.1
	48	43.0 ± 8.2	35.2 ± 4.3	31.3 ± 12.2	13.9 ± 9.8	61.4	74.3
PM (%)	0	64.0 ± 5.0	64.0 ± 4.2	26.4 ± 9.5	54.6 ± 6.4	82.2	69.0
	24	46.5 ± 7.5	42.9 ± 5.0	17.7 ± 11.2	18.7 ± 10.7	80.3	61.5
	48	37.1 ± 8.5	28.0 ± 4.2	25.5 ± 9.9	8.9 ± 7.8	53.1	97.4
VAP (μm/s)	0	85.4 ± 4.5	91.6 ± 4.1	83.7 ± 13.9	86.4 ± 11.7	100.0	40.2
	24	71.2 ± 5.8	80.9 ± 7.2	60.9 ± 19.9	49.2 ± 12.5	86.7	70.5
	48	138.8 ± 8.2	71.1 ± 4.5	81.8 ± 12.8	73.1 ± 2.8	65.3	97.4
VCL (μm/s)	0	145.7 ± 4.7	150.7 ± 5.2	143.9 ± 8.3	140.6 ± 0.8	164.4	67.2
	24	121.7 ± 8.5	139.6 ± 10.5	114.1 ± 41.7	91.8 ± 34.2	154.4	130.1
	48	63.1 ± 15.5	135.3 ± 7.9	151.6 ± 9.9	142.8 ± 1.0	126.2	162.9
VSL (μm/s)	0	67.7 ± 4.4	74.4 ± 4.3	66.2 ± 10.8	72.1 ± 9.5	81.1	33.6
	24	59.1 ± 4.5	67.2 ± 6.5	50.7 ± 16.8	39.5 ± 9.5	68.3	52.7
	48	63.1 ± 6.7	54.1 ± 3.1	61.3 ± 6.8	55.0 ± 1.5	51.0	81.4

Abbreviations: TM, total motility; PM, progressive motility; VAP, average path velocity; VCL, curvilinear velocity; VSL, straight-line velocity.

**Table 11:** Motility parameters of the first and second ejaculates of the season collected from breeding inactive stallions (n = 20) after 0, 24, and 48 h of cooling. The ejaculate was processed via cushion-centrifugation before cooling. Based on the motility parameters between the first and second ejaculates, sixteen stallions were classified as *unchanged* (i.e., ≤10% change), three stallions as *increased* (i.e., improvement > 10%) group, and one stallion as *decreased* (i.e., reduction > 10%).

		*Unchanged* (n = 16)		*Increased* (n = 3)		*Decreased* (n = 1)	
	Time (h)	First Ejaculate	Second Ejaculate	First Ejaculate	Second Ejaculate	First Ejaculate	Second Ejaculate
TM (%)	0	67.3 ± 4.1	63.7 ± 3.9	38.6 ± 14.5	54.8 ± 10.3	85.9	86.3
	24	61.2 ± 4.3	60.3 ± 5.8	29.8 ± 10.8	55.4 ± 9.0	88.3	76.8
	48	54.8 ± 5.3	63.4 ± 5.4	32.7 ± 9.1	29.5 ± 5.2	65.9	77.9
PM (%)	0	59.0 ± 4.1	53.9 ± 4.9	32.6 ± 13.8	48.9 ± 11.0	80.9	80.7
	24	54.7 ± 4.6	52.4 ± 5.7	24.3 ± 9.7	47.1 ± 7.4	80.7	73.7
	48	47.6 ± 5.6	56.4 ± 5.9	25.4 ± 6.8	22.3 ± 0.9	60.3	68.4
VAP (μm/s)	0	73.0 ± 6.3	75.6 ± 5.6	78.3 ± 19.2	77.4 ± 12.9	83.8	91.7
	24	70.7 ± 5.6	72.3 ± 5.3	77.2 ± 10.0	71.3 ± 4.0	80.3	77.1
	48	68.3 ± 5.5	70.8 ± 4.4	63.72.0	61.0 ± 2.4	75.3	83.8
VCL (μm/s)	0	134.3 ± 10.1	138.9 ± 9.2	135.1 ± 17.0	134.8 ± 8.0	159.9	171.1
	24	127.0 ± 6.7	125.8 ± 7.5	149.8 ± 9.0	133.4 ± 11.3	147.8	139.1
	48	125.1 ± 5.8	122.9 ± 5.3	123.1 ± 7.4	109.2 ± 4.7	134.7	146.7
VSL (μm/s)	0	57.0 ± 4.9	60.8 ± 4.6	62.2 ± 14.7	61.3 ± 12.2	62.6	72.2
	24	59.7 ± 5.4	59.6 ± 4.5	59.4 ± 3.1	56.6 ± 4.8	59.9	63.4
	48	56.5 ± 4.7	59.6 ± 4.0	51.3 ± 1.4	47.6 ± 2.4	61.9	67.1

Abbreviations: TM, total motility (%); PM, progressive motility (%); VAP, average path velocity; VCL, curvilinear velocity; VSL, straight-line velocity.

**Table 12:** Motility parameters and percentage of sperm with intact plasma membrane (PMI) and high mitochondrial membrane potential (HMMP) of the first and second ejaculates of the season collected from breeding inactive stallions (n = 20) before and after freezing.

	First Ejaculate		Second Ejaculate	
	Before Freezing	After Freezing	Before Freezing	After Freezing
TM (%)	64.6 ± 3.7 ^a^	51.7 ± 3.7 ^b^	65.6 ± 3.2 ^a^	50.4 ± 3.7 ^b^
PM (%)	57.0 ± 5.1 ^a^	44.4 ± 3.7 ^b^	56.9 ± 3.8 ^a^	39.8 ± 3.8 ^b^
VAP (μm/s)	72.8 ± 4.4 ^a^	60.6 ± 2.1 ^b^	80.2 ± 4.0 ^a^	72.5 ± 5.5 ^b^
VCL (μm/s)	135.6 ± 7.2 ^a^	109.1 ± 3.9 ^b^	142.4 ± 6.5 ^a^	95.9 ± 5.8 ^b^
VSL (μm/s)	56.7 ± 3.6 ^a^	48.6 ± 1.6 ^b^	64.6 ± 3.4 ^a^	40.3 ± 4.8 ^b^
PMI (%)	77.2 ± 4.0 ^a^	31.7 ± 4.3 ^b^	70.5 ± 4.6 ^a^	34.0 ± 5.0 ^b^
HMMP (%)	41.6 ± 2.8	45.9 ± 2.6	41.2 ± 3.7	53.3 ± 0.9

Abbreviations: PMI, plasma membrane integrity; HMMP, intact sperm with high mitochondrial membrane potential. Different superscripts (^ab^) denote a difference between columns (*p* < 0.05).

**Table 13:** Motility parameters of the first and second ejaculates of the season collected from breeding inactive stallions (n = 20) before and after freezing. Based on the motility parameters between the first and second ejaculates, sixteen stallions were classified as *unchanged* (i.e., ≤10% change), three stallions as *increased* (i.e., improvement > 10%) group, and one stallion as *decreased* (i.e., reduction > 10%).

**First Ejaculate**						
	***Unchanged* (n = 16)**		***Increased* (n = 3)**		***Decreased* (n = 1)**	
	**Before Freezing**	**After Freezing**	**Before Freezing**	**After Freezing**	**Before Freezing**	**After Freezing**
TM (%)	67.2 ± 3.8	52.7 ± 4.0	35.2 ± 13.6	31.0 ± 14.5	85.9	68.4
PM (%)	59.1 ± 3.8	44.2 ± 4.1	29.3 ± 13.3	25.6 ± 16.0	80.9	61.8
VAP (μm/s)	73.0 ± 5.0	57.2 ± 2.2	61.2 ± 1.5	62.3 ± 1.8	83.8	71.0
VCL (μm/s)	135.1 ± 8.0	103.2 ± 4.6	118.6 ± 1.5	118.8 ± 2.6	159.9	122.1
VSL (μm/s)	57.0 ± 4.0	45.6 ± 1.5	49.5 ± 1.0	50.5 ± 0.6	62.6	53.4
**Second Ejaculate**						
	***Unchanged* (n = 16)**		***Increased* (n = 3)**		***Decreased* (n = 1)**	
	**Before Freezing**	**After Freezing**	**Before Freezing**	**After Freezing**	**Before Freezing**	**After Freezing**
TM (%)	66.9 ± 3.1	51.2 ± 4.1	49.8 ± 5.5	39.0 ± 8.4	86.3	72.3
PM (%)	57.3 ± 4.1	41.7 ± 3.9	43.9 ± 6.3	21.8 ± 6.0	80.7	63.2
VAP (μm/s)	78.5 ± 4.8	72.5 ± 6.1	77.4 ± 12.9	72.8 ± 20.2	91.7	71.5
VCL (μm/s)	141.5 ± 8.0	97.2 ± 6.3	134.6 ± 8.0	79.2± 16.0	171.1	125.0
VSL (μm/s)	63.3 ± 3.9	41.6 ± 5.4	61.3 ± 12.2	28.4 ± 13.8	72.2	54.6

## Data Availability

Not applicable.
